# Fixation of Osteochondral Lesions of the Knee Using Autologous Bone Pegs: Functional Evaluation of a Case Series

**DOI:** 10.1055/s-0046-1822637

**Published:** 2026-06-08

**Authors:** Luis Henrique Longo, Anna Carolina Pavelec Costa, Camille Midori Okuyama, Edmar Stieven Filho, Marcos Paulo Tercziany Vanzin, Paulo Victor Oliveira Vieira de Souza

**Affiliations:** 1 Knee Surgery Division, Complexo Hospitalar do Trabalhador, Curitiba, PR, Brazil; 2 Hospital Universitário Evangélico Mackenzie, Curitiba, PR, Brazil; 3 Hospital de Clínicas, Universidade Federal do Paraná, Curitiba, PR, Brazil

**Keywords:** cartilage, articular, osteoarthritis, knee, transplantation, autologous, cartilagem articular, osteoartrite do joelho, transplante autólogo

## Abstract

**Objective:**

To evaluate the clinical and functional outcomes of patients who underwent fixation of traumatic osteochondral knee fractures using autologous bone pegs.

**Methods:**

We conducted a retrospective cross-sectional study including 11 patients who underwent surgery between 2021 and 2024. Clinical, radiographic, and functional outcomes were evaluated during a mean follow-up of 33.9 ± 10.6 months, including functional assessment using the International Knee Documentation Committee (IKDC) Subjective Knee Form.

**Results:**

The mean patient age was of 20.3 ± 5.4 years, with a predominance of patellar lesions (81,8%). The radiographic evaluation demonstrated consolidation of the osteochondral fragment in all cases, without signs of displacement or fixation failure. The mean IKDC score was of 75.0 ± 17.1 points. There were no major complications or reoperations.

**Conclusion:**

Fixation of traumatic osteochondral knee fractures with autologous bone pegs resulted in adequate consolidation and satisfactory functional outcomes in this case series, representing a safe, effective, and low-cost biological alternative to manage these lesions.

## Resumo

**Objetivo:**

Avaliar os desfechos clínico-funcionais de pacientes submetidos à fixação de fraturas osteocondrais traumáticas do joelho com palitos ósseos autólogos.

**Métodos:**

Realizamos um estudo retrospectivo transversal, que incluiu 11 pacientes operados entre 2021 e 2024. Foram analisados dados clínicos, radiográficos e funcionais, com aplicação do Formulário de Avaliação Subjetiva do Joelho do International Knee Documentation Committee (IKDC) durante o acompanhamento ambulatorial médio de 33,9 ± 10,6 meses.

**Resultados:**

A amostra apresentou média etária de 20,3 ± 5,4 anos, com predomínio de lesões patelares (81,8%). A avaliação radiográfica demonstrou consolidação do fragmento osteocondral em todos os casos, sem sinais de deslocamento ou falha da fixação. A pontuação média no IKDC foi de 75,0 ± 17,1. Não foram observadas complicações graves ou necessidade de reintervenções cirúrgicas.

**Conclusão:**

A fixação de fraturas osteocondrais traumáticas do joelho com palitos ósseos autólogos resultou em consolidação adequada e desfechos funcionais satisfatórios nesta série de casos, e configura-se como alternativa biológica segura, eficaz e de baixo custo para o manejo dessas lesões.

## Introduction

Osteochondral fractures of the patella and femoral condyle, although uncommon, 1 represent a therapeutic challenge due to their limited healing potential. 2 These lesions occur predominantly in young, physically-active subjects, and they are typically associated with acute trauma—most commonly patellar dislocation—or secondary conditions such as osteochondritis dissecans. 3 The optimal management of these fractures remains a subject of debate, 2 4 as there is no consensus regarding the most effective surgical technique. Nevertheless, preservation of the articular cartilage remains the primary therapeutic goal, since its deterioration or the formation of loose bodies may lead to significant complications, including functional limitation and early osteoarthritis. 2 5 6

Among the surgical techniques described for fixation of osteochondral fragments, the use of autologous bone pegs has emerged as a biologically-advantageous alternative. 2 7 8 9 10 This approach, first described by Bandi and Allgoewer 11 in 1959, involves the use of bone grafts harvested from the ipsilateral tibial metaphysis, which are inserted into the osteochondral fragment to promote stabilization and osseous integration.

Bone pegs, as autologous tissues, favor biological integration with the recipient site and eliminate the need for synthetic implants. This technique also avoids the use of permanent hardware, thereby reducing the risk of inflammatory reactions. 2 7 10 In contrast, fixation with metallic screws often requires subsequent removal, and it may cause damage to the adjacent cartilage. 1 12 13 Bioabsorbable implants have also been used for osteochondral fixation; however, their use remains limited and may be associated with foreign-body reactions. 4 14 15 16

Despite these advantages, there is a lack of clinical studies 2 17 evaluating the long-term functional outcomes of patients treated with autologous bone pegs. Therefore, the present study aimed to evaluate the clinical and functional outcomes of patients with traumatic osteochondral knee lesions treated with this technique.

## Materials and Methods

The current retrospective cross-sectional study was conducted at a tertiary trauma center, and it included patients who underwent fixation of osteochondral knee fractures through open reduction and internal fixation using autologous bone pegs between January 2021 and January 2024. The Institutional Research Ethics Committee approved the study under CAAE: 87552425.7.0000.5225.

We identified patients who underwent fixation of osteochondral knee lesions using autologous bone pegs during the study period. The inclusion criteria were a diagnosis of traumatic osteochondral knee fracture with a fragment suitable for fixation, a minimum outpatient follow-up of 12 months, and skeletal maturity confirmed by radiographs demonstrating closed physes.

The exclusion criteria were prior surgery on the affected knee, skeletal immaturity, comminuted or non-viable fragments for fixation, and non-traumatic osteochondral lesions (such as osteochondritis dissecans).

Initially, 13 patients were considered eligible. One patient was excluded due to a history of surgery on the affected knee, and another, due to a diagnosis of osteochondritis dissecans, resulting in a final sample of 11 patients.

Preoperatively, all patients underwent clinical evaluations, radiographs, and computed tomography (CT) and magnetic resonance imaging (MRI) scans. The diagnosis of traumatic osteochondral fractures relied on the identification of a displaced fragment, with involvement of the subchondral bone, confirmed through CT and MRI scans, which enabled its differentiation from purely chondral lesions that do not have an associated osseous component.

Computed tomography was primarily used to assess fragment morphology, size, and viability, while MRI enabled the evaluation of the articular cartilage, subchondral bone, and associated injuries. Next, all patients were referred for surgical treatment by the same knee surgeon.

Data was obtained through a retrospective review of electronic medical records and in-person outpatient evaluations conducted by other team members. The variables analyzed included age, gender, affected side, injury etiology, lesion size, fracture location, time from injury to surgery, number of bone pegs used, time until the postoperative evaluation, presence of complications, and functional outcome as measured by the International Knee Documentation Committee (IKDC) Subjective Knee Form. 18 Due to the acute nature of the cases, the IKDC score was not systematically collected preoperatively, representing a limitation to the current study.

**Fig. 1:**
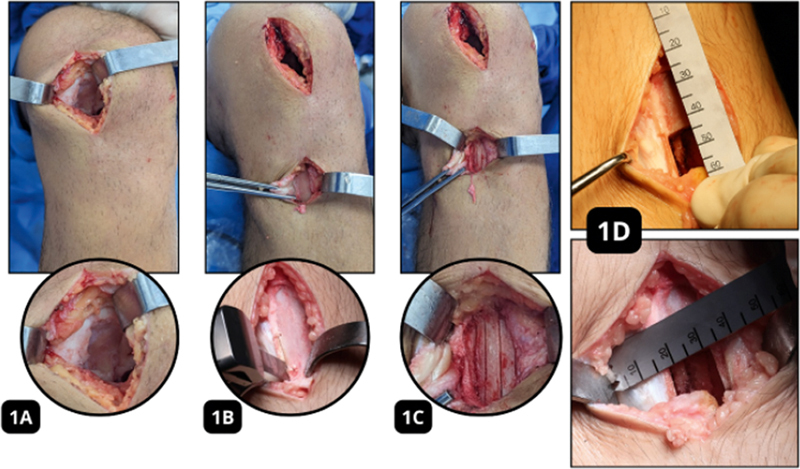
Sequence of osteochondral lesion exposure and segmentation of the proximal anteromedial metaphyseal cortical bone.

All procedures began with arthroscopic inspection for identification and careful removal of the osteochondral fragment. The lesion bed was then exposed through a medial or lateral parapatellar approach, depending on fracture location, enabling direct visualization of the affected area ( Fig. 1 ).

A segment of the ipsilateral, proximal, anteromedial tibial metaphysis was harvested using a fine oscillating saw measuring approximately 20 mm in length and 15 to 16 mm in width. Parallel and equidistant longitudinal cuts were then made to divide the anteromedial cortical bone into regular segments of approximately 20 × 4 mm, facilitating subsequent preparation of the bone pegs ( Fig. 1 ).

**Fig. 2:**
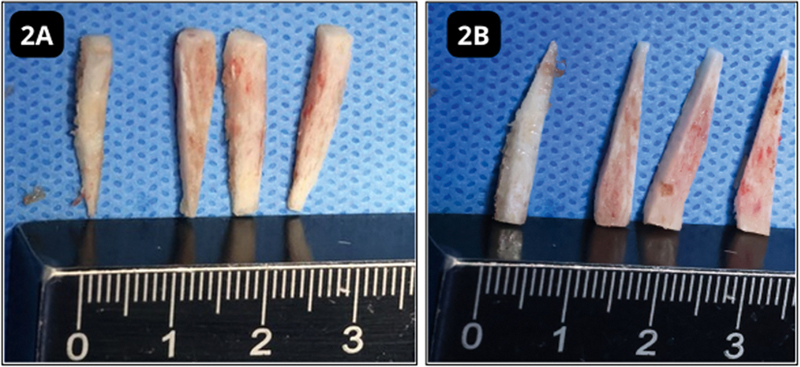
Prepared autologous bone pegs.

The bone pegs were further shaped on the surgical table using osteotomes or a scalpel, resulting in cylindrical, arrow-shaped grafts measuring approximately 20 mm in length, with a diameter of 2 to 3 mm at the tip and 3 to 4 mm at the base, suitable for press-fit fixation within the lesion bed ( Fig. 2 ).

**Fig. 3:**
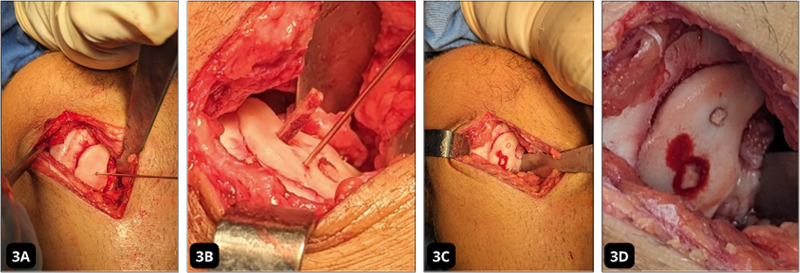
Fixation of the osteochondral lesion using autologous bone pegs and final construct appearance.

After preparation of the lesion bed and repositioning of the osteochondral fragment, provisional stabilization was achieved using a Kirschner wire. This was followed by predrilling with a fine drill or a 2.0-mm Kirschner wire. The bone pegs were then inserted by press-fit impaction through the prepared holes using a surgical mallet and graft impactor. A minimum of three bone pegs was used per lesion, ensuring that their ends were flush with the subchondral bone without protruding into the articular surface. Fixation was performed by first placing a central peg, followed by peripheral pegs with equidistant distribution to ensure rotational and axial stability ( Fig. 3 ).

Associated procedures, such as medial patellofemoral ligament (MPFL) reconstruction, were performed in patients with objective patellar instability (documented prior dislocation) associated with the fracture.

Postoperative knee immobilization with an inguinomalleolar brace lasted for the first 2 weeks. Next, the patients resumed passive knee mobilization, initially limited to a range of motion from 0 to 60°, with gradual progression to full flexion/extension between the fourth and sixth weeks. In cases of femoral-condyle lesions, complete non-weight-bearing was recommended for 6 weeks, followed by gradual progression to full weight-bearing by the 12 ^th^ week. Patients with patellar lesions were not allowed to bear weight for 2 weeks. Return to activities of daily living was allowed gradually in approximately 3 months, according to individual clinical progression. Serial radiographs assessed fracture consolidation, defined by the absence of a visible fracture line and integration of the fragment into the bone bed in at least two radiographic views. Follow-up MRI scans were not routinely performed, being reserved for patients with atypical clinical progression.

The patients were followed regularly in the outpatient setting according to the institutional protocol, with discharge typically occurring around 12 months postoperatively. For the present study, patients who underwent surgery in earlier periods were recalled for reassessment, during which they received detailed information about the research and provided written informed consent. At this time, the IKDC form was applied once for each patient, not necessarily at the same postoperative time point across subjects, characterizing a cross-sectional analysis.

Data was organized using electronic spreadsheets (Microsoft Excel, Microsoft Corp.) and analyzed descriptively. The quantitative variables were expressed as mean, standard deviation, or median values, depending on the distribution. The categorical variables were presented as absolute and relative frequencies.

## Results

A total of 11 patients, with a mean age of 20.3 ± 5.4 years, who underwent fixation of traumatic osteochondral knee fractures using autologous bone pegs were evaluated. Most patients were male (63.6%; n = 7), and the left knee was more frequently affected (63.6%; n = 7). Regarding etiology, 8 patients (72.7%) presented patellofemoral instability, while 3 patients (27.3%) sustained fractures due to direct trauma.

The most common fixation site was the patella (n = 9; 81.8%), followed by the lateral femoral condyle (n = 2; 18.2%). The mean size of the osteochondral lesions was of 3.4 ± 1.1 cm ^2^ , with an average of 4.1 ± 1.6 bone pegs used per procedure. In 3 cases (27.3%), additional procedures were performed during the same surgical session, including 3 MPFL reconstructions and 1 lateral retinacular release.

The mean time from injury to surgery was of 3.5 ± 1.0 days. The mean postoperative follow-up was of 33.9 ± 10.6 months. During this period, there were 3 complications, including 2 patients who developed joint stiffness requiring manipulation under anesthesia to restore range of motion, and 1 patient who presented with complex regional pain syndrome. Table 1 summarizes lesion characteristics, intraoperative data, and outpatient follow-up details.

**Table 1:** Characteristics of osteochondral lesions, intraoperative data, and follow-up

Patient (age in years)	Fixation site	Time from injury to surgery (days)	Lesion size (cm ^2^ )	No. of bone pegs	Concomitant procedure	Complications	Follow-up (months)
1 (19)	Patella	3	3.0	3	No	No	47
2 (19)	Patella	4	3.8	7	No	No	44
3 (18)	Patella	5	4	5	No	No	44
4 (20)	Patella	3	2.2	3	No	Joint stiffness	44
5 (27)	LFC	2	4	6	No	No	43
6 (29)	Patella	2	3.5	6	MPFLR + LRR	No	41
7 (25)	LFC	3	3	3	MPFLR	Joint stiffness/CRPS	30
8 (18)	Patella	5	2.5	3	No	No	24
9 (26)	Patella	3	2.5	3	No	No	21
10 (13)	Patella	4	2	3	No	No	18
11 (15)	Patella	4	0.8	2	MPFLR	No	17

**Abbreviations:** LFC, lateral femoral condyle; LLR, lateral retinacular release; MPFLR, medial patellofemoral ligament reconstruction; CRPS, complex regional pain syndrome.

**Fig. 4:**
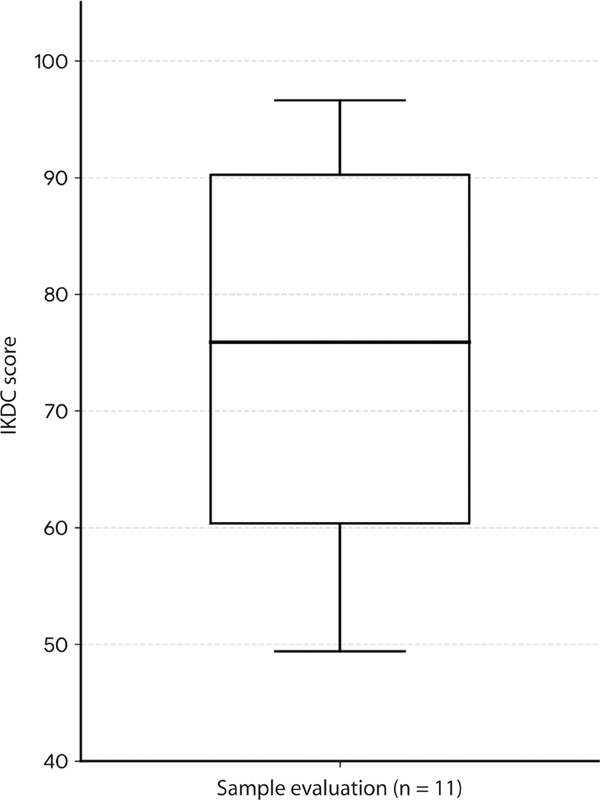
Boxplot of the scores on the International Knee Documentation Committee (IKDC) Subjective Knee Form.

The functional outcomes were assessed using the IKDC form, which includes three main domains: symptoms, knee function, and level of sports activity. The mean IKDC score was of 75.0 ± 17.1 points, with a median of 75.9 points ( Fig. 4 ).

The functional evaluation using the IKDC form demonstrated satisfactory clinical outcomes across all three domains. In the symptomatic domain, the median pain levels were low (1.5 out of 10), with most patients reporting absent or mild stiffness and swelling. In terms of overall knee function, the mean score of 75.0 ± 17.1 (maximum value: 96.0) points suggests satisfactory recovery in most cases. Regarding sports activity, more than half of the patients returned to moderate activities, and a subset reported tolerance to vigorous exercise. Appendix 1 presents detailed data.

## Discussion

Although described as a promising biological alternative, fixation of osteochondral lesions using autologous bone pegs remains underexplored in the literature, being more frequently reported in cases of osteochondritis dissecans 10 19 20 or in isolated reports of osteochondral fractures treated with this technique. 7 8 In this context, the present study aimed to describe a case series of traumatic osteochondral knee fractures treated with autologous bone pegs, with functional evaluation over a mean follow-up of approximately 3 years.

In the present study, most patients demonstrated satisfactory functional recovery, with a mean score on the IKDC form of 75.0 ± 17.1 and a high rate of return to moderate or vigorous physical activity. Subjective perception of knee function remained stable, with mean scores of 8.8 ± 1.3 in the preinjury period and of 8.6 ± 1.4 postoperatively, with a median of 9.0 at both time points. These findings are consistent with those reported by Ogura et al., 2 who described an 83% success rate in 6 adolescents (mean age: 12.9 years) treated with fixation of pure chondral fragments using autologous bone pegs, with a mean follow-up of 5.2 (range: 1.4–10.9) years. In that study, patients achieved full return to sports at a mean time of 7 months, and they presented a mean Magnetic Resonance Observation of Cartilage Repair Tissue (MOCART) score of 85 (range: 70–95) points, indicating adequate cartilage healing on MRI scans. Despite differences in patient populations, both studies demonstrate satisfactory functional recovery, preservation of articular congruency, and the favorable biological integration potential provided by autologous bone peg fixation.

The positive outcomes observed in the present study may be associated with the age profile of the sample. The patients included were young, with a mean age of 20.3 years, an age group in which a better prognosis is expected for chondral and osteochondral lesions. In a previous study 21 conducted by our group, age was identified as a key prognostic factor in the treatment of osteochondral lesions, with patients older than 40 years of age presenting worse clinical and functional outcomes. Thus, the favorable results observed in the current series may also reflect the superior biological healing capacity of younger subjects.

Two complications occurred among the patients included in the cpresent study: 2 patients developed joint stiffness, 1 of whom also presented with complex regional pain syndrome (CRPS). In both cases, manipulation under anesthesia was performed, followed by an intensive rehabilitation protocol including physiotherapy and muscle strengthening, with satisfactory recovery and no need for further surgical intervention. The case of CRPS was managed clinically with analgesics and pain-modulating medications. No cases of infection, loose body formation, or need for removal of the bone pegs were recorded.

From a biomechanical perspective, the experimental study by Sasaki et al. 22 compared three fixation methods in an animal model—autologous bone pegs, bioabsorbable pins, and suture anchors with 2-0 tape. The bone pegs demonstrated initial strength compatible with intra-articular demands, although lower than that observed in the anchor group. Importantly, no statistically significant differences were found among groups regarding the load required to produce 1 mm of displacement, a critical parameter of primary stability. These findings are consistent with the results of the current study, since the technique demonstrated adequate stability and no mechanical failures during follow-up.

Compared with other fixation techniques, transosseous suture represents an alternative that also avoids the use of prominent implants. Studies employing this approach for patellar osteochondral fractures have reported favorable clinical outcomes and high union rates. 23 24 From a biomechanical standpoint, the main advantage of autologous bone pegs over suture fixation may lie in greater compressive and rotational stability of the fragment, particularly in larger lesions or those with a thicker subchondral bone layer. Conversely, transosseous suture may be preferable in fragments with thinner subchondral bone, in which insertion of bone pegs becomes technically more challenging. Therefore, the choice of fixation technique should be individualized, considering the characteristics of the osteochondral fragment, the quality of the subchondral bone, and the surgeon's experience.

Despite the favorable results reported with metallic implants and bioabsorbable devices for osteochondral fracture fixation, 4 13 25 these techniques are not without complications. The reported disadvantages include the need for secondary surgery for hardware removal, risk of damage to adjacent cartilage, inflammatory reactions to the implant material, and the possibility of nonunion or inadequate fragment healing. 1 4 12 13 14 16 25 In contrast, fixation with autologous bone pegs eliminates the risk of implant-related inflammatory response, avoids the need for removal, and preserves cartilage integrity, representing a biological and cost-effective alternative.

Despite the biological advantages of this technique, some operative limitations should be considered. Bone peg preparation requires harvesting a segment from the proximal tibial metaphysis, which may result in local pain, hematoma, and, rarely, fracture. 19 Additionally, this is a technique-dependent procedure that requires precision both in graft preparation and intra-articular placement, with potential risks of fragment instability or articular incongruity if not performed adequately.

**Fig. 5:**
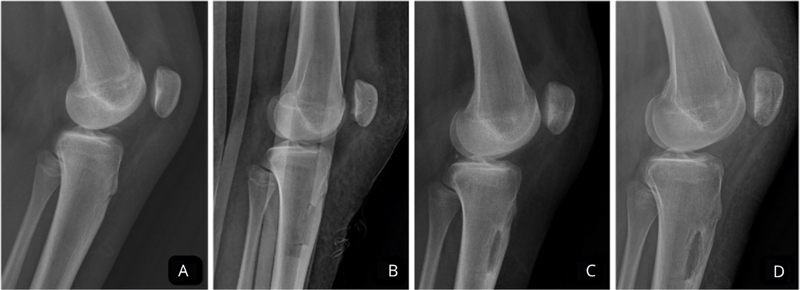
Radiographic progression demonstrating consolidation of a patellar osteochondral lesion. **A** ) Radiograph upon admission; ( **B** ) immediate postoperative radiograph (with brace); ( **C** ) 3-month follow-up; and ( **D** ) 6-month follow-up.

In the current series, clinical and radiographic evidence of satisfactory osseous integration was observed in all cases ( Fig. 5 ), typically occurring between 3 and 6 months postoperatively, with no cases of nonunion or need for reintervention. Although not all patients underwent follow-up MRI or assessment with specific scoring systems such as the MOCART, 26 the available data suggest adequate incorporation of the osteochondral fragment. Imaging identified areas of subchondral sclerosis in three cases, a finding previously described as a possible consequence of techniques involving subchondral bone drilling for implant insertion. 2 27 28 Up to the last follow-up, these changes did not demonstrate clinical relevance. Nevertheless, the importance of long-term clinical and radiographic follow-up should be emphasized, as subchondral alterations may, in other contexts, be associated with failure of integration or late cartilage degeneration. 26

Among the limitations of the present study, the case series design is the primary factor restricting generalizability. The lack of systematic preoperative IKDC assessment precludes objective quantification of functional improvement. Although all patients showed satisfactory radiographic consolidation, some did not undergo advanced imaging, such as MRI scans, which limits the consistent use of specific scoring systems such as the MOCART. 26 The absence of a control group treated with an alternative fixation technique also hinders direct comparisons between approaches. Lastly, although the follow-up period is adequate for initial functional assessment, it remains insufficient to detect potential late subchondral changes. Future studies with larger sample sizes, longer follow-up, and standardized functional and radiological assessments are essential to validate and expand upon these findings.

## Conclusion

Fixation of osteochondral knee fractures using autologous bone pegs proved to be an effective technique, resulting in consistent osseous consolidation and favorable functional outcomes in the current case series. This procedure represents a low-cost biological alternative that avoids complications associated with metallic or bioabsorbable implants. The findings suggest that this technique is a safe and viable treatment option for young patients with this type of lesion.
